# Interleukin-6 overproduction correlates with disease activity and severity in juvenile idiopathic arthritis

**DOI:** 10.1186/1546-0096-9-S1-P109

**Published:** 2011-09-14

**Authors:** Mihaela Spirchez, Gabriel Samasca, Claudia Bolba, Nicolae Miu

**Affiliations:** 1 Department of Pediatrics, 2nd Pediatric Clinic, „Iuliu Ha?ieganu” University of Medicine and Pharmacy, Cluj-Napoca, Romania; 2 Department of Immunology, „Iuliu Ha?ieganu” University of Medicine and Pharmacy, Cluj-Napoca, Romania

## Background

IL-6 has long been known to play the key role in mediating the inflammatory process in a number of autoimmune diseases, including Juvenile Idiopathic Arthritis (JIA).

## Aim

To evaluate the possible role of IL-6 in monitoring disease activity in JIA.

## Methods

In a 2-year prospective study, IL-6 levels were measured using ELISA in 63 serum samples for 40 JIA patients. The control population consisted of 18 healthy children. The data were correlated with disease activity and severity (quantified with JADAS27 score) and also with several biomarkers of inflammation.

## Results

The patients with active disease had greater IL-6 levels than did the patients with inactive disease (p=0.002) and controls (p=0.006).The cutoff value for active disease obtained from the ROC curve was 8,33pg/ml. During the active stage of the disease IL-6 levels were significantly higher in both systemic and polyarticular patients than in the oligoarticular patients.

Serum IL-6 concentrations of active oligoarticular patients were not significantly different from those of controls (p=0.078). Levels of circulating IL-6 were elevated in patients with severe and moderate disease activity (JADAS27>10) compared with those of low disease activity (JADAS27≤10) (p=0.010) and controls (p=0.002). In our patients the circulating IL-6 values were significantly correlated with JADAS27 score (r=0.49; p<0.01), ESR (r=0.38; p<0.01), CRP (r=0.33; p=0.01) and with hemoglobin levels (r= -0.30; p=0.02).

## Conclusions

Serum IL-6 concentrations may serve as a biomarker of disease activity and severity in JIA. Our data are also encouraging for the clinicians to focus on IL-6 as a target for therapeutic inhibition.

